# Severe Vitamin A Deficiency After Biliopancreatic Diversion

**DOI:** 10.1177/2324709619888051

**Published:** 2019-11-11

**Authors:** Lauren M. Lemieux, Vijaya Surampudi

**Affiliations:** 1 Department of Medicine, Division of Clinical Nutrition, University of California, Los Angeles, CA, USA

**Keywords:** biliopancreatic diversion, bariatric surgery, vitamin A, malabsorption

## Abstract

Biliopancreatic diversion is a surgical procedure that causes weight loss via volume restriction and malabsorption. It is now rarely performed due to the risk of severe nutritional deficiencies including vitamin A. We report a case of severe vitamin A deficiency due to malabsorption from a biliopancreatic diversion procedure for weight loss. By the time the patient presented to our department, she had developed blindness refractory to parenteral vitamin A treatment. A unique feature of her case is the development of a rash with vitamin A injections. This reaction has only been reported in one case series of 3 patients in the published literature. Her case highlights the importance of vitamin deficiency screening in patients after bariatric surgery, and her skin reaction to the injections is a unique side effect that is not frequently observed.

## Introduction

**Figure 1.**
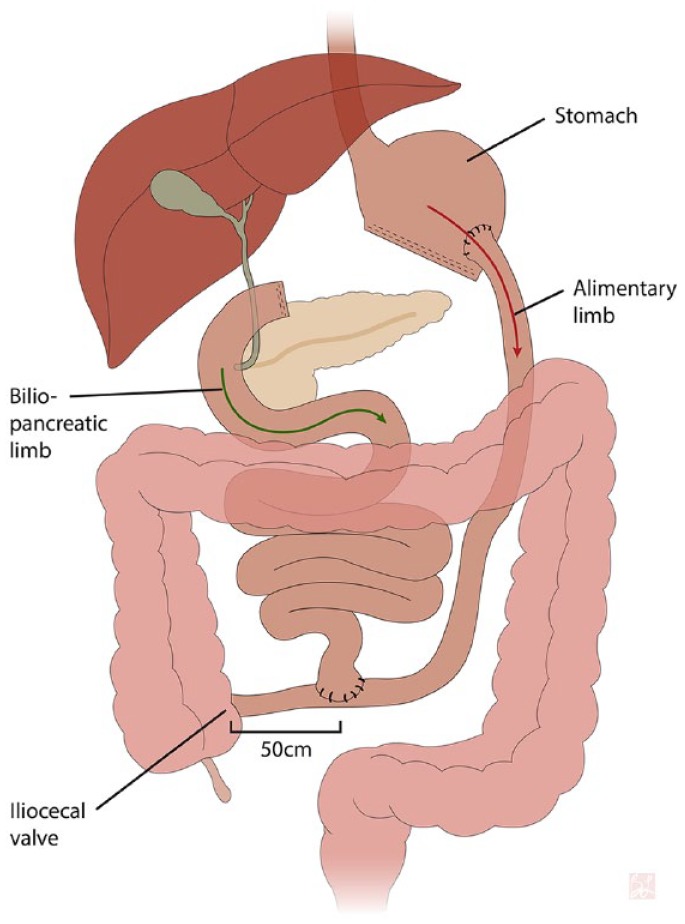
Biliopancreatic diversion schematic. Lemieux, B 2019.

Bariatric surgery is one of the fastest-growing operative procedures performed worldwide. It is unique in that it can potentially cure numerous diseases including diabetes, fatty liver disease, hypertension, sleep apnea, and many more. The various procedures involve volume restriction and/or nutrient malabsorption to achieve and sustain weight loss. One of the operations known as the biliopancreatic diversion (BPD) or the Scopinaro procedure (Figure 1) is now rarely performed due to the high risk of severe malnutrition and micronutrient deficiencies including, in particular, vitamin A deficiency.^1^

A study of 376 patients who had undergone BPD with or without duodenal switch found that 1 year after surgery vitamin A levels were low in 52% of patients and after 4 years this increased to 69% of patients, despite compliance to vitamin supplementation.^2^ The earliest and most common symptom of vitamin A deficiency is night blindness.^3^ Vitamin A deficiency can also cause other ophthalmologic disorders including xeropthalmia, keratomalacia, retinopathy, and blindness.^1,4^ Early stages of vitamin A deficiency may be reversed by oral or parenteral supplementation,^4,5^ but later stages can have varying response to vitamin A supplementation.^6,7^

## Case

A 68-year-old female with a history of BPD surgery in 1987 was referred for treatment of vitamin A deficiency. Her symptoms began with poor vision at nighttime but eventually progressed to blindness in her left eye. At the time of her diagnosis in 2013, her vitamin A level was 4 µg/dL (normal = 38-98 µg/dL), and she already had findings of retinal degeneration. She was initially treated with oral vitamin A supplementation of 100,000 IU daily; however, levels did not normalize consistently, and her retinopathy progressed. On referral to our division, she was started on intramuscular vitamin A injections in June 2018. Prior to starting intramuscular vitamin A, her vitamin A level was 20 µg/dL (normal = 30-90 µg/dL).

**Figure 2.**
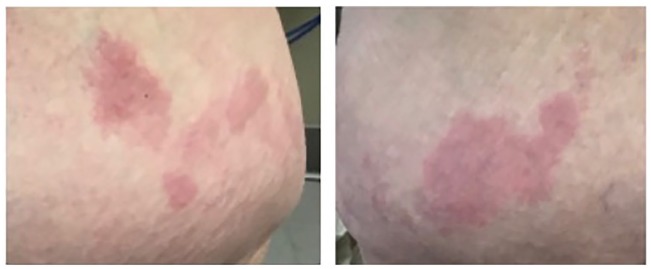
Vitamin A palmitate injection sites with overlying rash.

After receiving the first injection, 12 to 15 hours later she developed redness and scaling at site of injection. The second injection caused a similar reaction. Rashes seen at the injection sites are shown in Figure 2. The rash was not associated with any systemic symptoms, facial, tongue, throat swelling, or hive-like reaction. The etiology of the rash was thought to be an injection site reaction versus type IV hypersensitivity reaction. Although less likely, an irritant dermatitis was considered where the injection solution was coming in contact with the skin given the finding of one injection site without associated rash. The suspicion for a type I hypersensitivity was low given the delayed onset of the rash, and the patient declined skin prick testing for definitive evaluation. Overtime, her vitamin A levels improved but her vision did not.

## Discussion

Although less frequently performed today, BPD can lead to severe nutrient deficiencies. In our patient’s case, treatment for vitamin A deficiency was not initiated until after the patient had irreversible retinopathy. Therefore, it is important to routinely screen patients for nutrient deficiencies before they develop. The American Society for Metabolic and Bariatric Surgery nutritional guidelines recommends screening for vitamin and mineral deficiencies every 3 to 6 months during the first 2 years and annually thereafter. For patients who have had a Roux-en-Y gastric bypass or BPD, this includes measuring serum levels of vitamins A, B_1_, B_12_, D, and folate as well as zinc, copper, and iron studies.^8^ Rash in the setting of vitamin A injections has only been reported in one case series of 3 patients.^9^ In this case series, patients underwent skin testing and only reacted to polysorbate 80, an emulsifier found in injectable vitamin A palmitate, but not to retinol palmitate or other vitamin A injection constituents (hydroxyanisole, hydroxytoluene, chlorobutanol, and citric acid).^9^ Polysorbate 80 is also used in other parental medications and some vaccines.^9^ Rash following vitamin A palmitate is an uncommon side effect, but it should be closely monitored for progressive allergic reaction and patients should be aware of potential reactions to vaccines and other medications that contain similar components.
